# High parasite virulence necessary for the maintenance of host outcrossing via parasite-mediated selection

**DOI:** 10.1093/evlett/qrad036

**Published:** 2023-09-05

**Authors:** Samuel P Slowinski, JaeHoon Cho, McKenna J Penley, Laura W Alexander, Arielle B Greenberg, Sathvik R Namburar, Levi T Morran

**Affiliations:** Department of Biology, University of Maryland, College Park, MD, United States; Department of Biology, Emory University, Atlanta GA, United States; Department of Biology, Emory University, Atlanta GA, United States; Department of Integrative Biology, University of California, Berkeley, CA, United States; Department of Biology, Emory University, Atlanta GA, United States; Department of Biology, Emory University, Atlanta GA, United States; Department of Biology, Emory University, Atlanta GA, United States

**Keywords:** experimental evolution, virulence, Red Queen hypothesis, coevolution, parasite, sex

## Abstract

Biparental sex is widespread in nature, yet costly relative to uniparental reproduction. It is generally unclear why self-fertilizing or asexual lineages do not readily invade outcrossing populations. The Red Queen hypothesis predicts that coevolving parasites can prevent self-fertilizing or asexual lineages from invading outcrossing host populations. However, only highly virulent parasites are predicted to maintain outcrossing, which may limit the general applicability of the Red Queen hypothesis. Here, we tested whether the ability of coevolving parasites to prevent invasion of self-fertilization within outcrossing host populations was dependent on parasite virulence. We introduced wild-type *Caenorhabditis elegans* hermaphrodites, capable of both self-fertilization and outcrossing, into *C. elegans* populations fixed for a mutant allele conferring obligate outcrossing. Replicate *C. elegans* populations were exposed for 24 host generations to one of four strains of *Serratia marcescens* parasites that varied in virulence, under three treatments: a heat-killed (control, noninfectious) parasite treatment, a fixed-genotype (nonevolving) parasite treatment, and a copassaged (potentially coevolving) parasite treatment. As predicted, self-fertilization invaded *C. elegans* host populations in the control and fixed-parasite treatments, regardless of parasite virulence. In the copassaged treatment, selfing invaded host populations coevolving with low- to mid-virulence strains, but remained rare in hosts coevolving with highly virulent bacterial strains. Therefore, we found that only highly virulent coevolving parasites can impede the invasion of selfing.

## Introduction

Biparental outcrossing is the dominant mode of reproduction among plants and animals. Less than 1% of animal species are obligately asexual, and few plant and animal species reproduce by obligate self-fertilization (Bell, 1982; Goodwillie et al., 2005; Jarne & Auld, 2006; Judson & Normark, 1996). This finding is surprising because substantial inherent costs are associated with biparental mating systems (Bell, 1982; Gibson et al., 2017; Lively & Lloyd, 1990; Maynard Smith, 1978; Williams, 1975). For example, a population that relies on uniparental reproduction, like self-fertilization or parthenogenesis, does not bear the “two-fold cost of males” (Maynard Smith, 1978) typical of obligately outcrossing populations. This cost renders obligately outcrossing populations potentially susceptible to invasion and replacement by individuals with mutant alleles conferring uniparental reproduction (Agrawal & Lively, 2001; Charlesworth, 1980; Fisher, 1941; Maynard Smith, 1978). However, the widespread and long-term maintenance of obligate outcrossing suggests that biparental reproduction confers sufficient advantages over uniparental reproduction to outweigh the inherent costs of outcrossing.

The Red Queen hypothesis (RQH) for the maintenance of sex by coevolving parasites predicts that coevolution with parasites can maintain outcrossing in host populations. It proposes that coevolving parasites select against common host genotypes and favor host outcrossing over self-fertilization because outcrossing has the capacity to generate genetically variable offspring (Hamilton, 1980; Jaenike, 1978). Empirical studies of both natural and laboratory host populations have supported this prediction. Studies on natural snail populations in New Zealand, for example, have shown that frequencies of trematode parasite infection were positively correlated with rates of host sexual reproduction across lakes and streams and across multiple sites within lakes (King et al., 2011; Lively, 1987; Vergara et al., 2013). Furthermore, laboratory experiments on mixed-mating (capable of both self-fertilization and outcrossing) *Caenorhabditis elegans* nematode hosts found that exposure to nonevolving bacterial parasites favored the evolution of short-term increases in outcrossing rates (Morran et al., 2009) and that exposure to coevolving bacterial parasites selected for the maintenance of elevated outcrossing rates (Morran et al., 2011). Additional experiments have also shown that host adaptation to coevolving parasites was positively correlated with host outcrossing rates (Morran et al., 2011, 2013; Penley et al., 2017). Furthermore, Slowinski et al. (2016) directly tested the ability of coevolving parasites to impede the invasion of self-fertilization into *C. elegans* host populations fixed for a mutant allele conferring obligate outcrossing. As predicted, selection imposed by coevolving parasites impeded the invasion of self-fertilization, while self-fertilization invaded host populations under selection from a fixed-genotype (nonevolving) parasite and those exposed to a heat-killed (control) parasite. Therefore, coevolving parasites, but not nonevolving parasites, have been demonstrated to maintain host outcrossing.

While the RQH has empirical support as a potential mechanism for the maintenance of biparental sex, evolutionary theory predicts that the overall generalizability of the RQH may be limited. Specifically, coevolving parasites are only predicted to maintain host outcrossing under conditions in which parasite virulence is very high. Theoretical models by Otto and Nuismer (2004) predicted that, under most circumstances, species interactions do not select for sex. Given the limitations that biologically relevant parameters impose, they argued that the RQH alone could not explain the prevalence of outcrossing in nature. Additional theoretical models by Howard and Lively (1998) also predicted that coevolutionary interactions with parasites can select for host sex, but only when parasite virulence is moderate or high. Because the parasite (*S. marcescens* strain Sm2170) used in the studies by Morran et al. (2011) and Slowinski et al. (2016) is highly virulent (~90% host mortality) and capable of imposing strong selection against nematode hosts, their results leave open the question as to whether low- to moderately-virulent coevolving parasites can maintain host outcrossing.

Parasite virulence varies significantly in nature, but theory predicts many parasites to have low to intermediate levels of virulence (Bonhoeffer et al., 1996; Carval & Ferriere, 2010; Lenski & May, 1994), which may not always meet the threshold level of virulence predicted to maintain host outcrossing (Howard & Lively, 1998). Therefore, the RQH may be limited in scope as an explanation for the widespread maintenance of outcrossing. Here, we tested the prediction that the ability of parasites to prevent self-fertilization from spreading into outcrossing host populations is contingent on high parasite virulence. We utilized a similar experimental design to that described in Slowinski et al. (2016); we introduced an allele conferring mixed mating (*fog-2(wt)*, hereafter the mixed-mating allele) into a population of obligately outcrossing *C. elegans* hosts fixed for the mutant allele *fog-2(q71)*. The *fog-2(q71)* allele (hereafter the obligate-outcrossing allele) blocks the production of viable sperm in hermaphrodites, functionally transforming hermaphrodites into females (Schedl & Kimble, 1988). The obligate-outcrossing allele has no known effects on the male phenotype (Schedl & Kimble, 1988). Hermaphrodites (harboring at least one mixed-mating allele) and females (harboring two obligate-outcrossing alleles) are all capable of outcrossing with any male, regardless of the male’s *fog-2* genotype (Schedl & Kimble, 1988). Therefore, the mixed-mating genotypes and the obligately outcrossing genotypes can freely intermix.

We then exposed the hosts to four different *S. marcescens* strains that vary in virulence. *S. marcescens* is a bacterial parasite that can be highly virulent in *C. elegans*. *C. elegans* can exhibit both resistance and avoidance (Penley & Morran, 2018), and there is a genetic basis to *S. marcescens* infectivity and host resistance (Morran et al., 2011, 2013, 2014). On each of the four *S. marcescens* strains, hosts were passaged in three different treatments: heat-killed (control) parasites, fixed-genotype (nonevolving) parasites, or parasites copassaged (potentially coevolving) with the hosts. Overall, we predicted that copassaged parasites would constrain the invasion of self-fertilization into host populations, but only in host populations exposed to highly virulent parasite strains.

## Methods

### Establishment of obligately outcrossing and mixed-mating *C. elegans* host populations

#### Obligately outcrossing C. elegans populations

An obligately outcrossing *C. elegans* population (designated as CF3) was established by and is further detailed in Slowinski et al. (2016). In short, the obligately outcrossing *C. elegans* strain PX386 was first generated by backcrossing the obligate-outcrossing allele, *fog-2* (*q71*), into an inbred strain of CB4856 (PX382). One thousand PX386 individuals were then exposed to 40 mM ethyl methanesulfonate (EMS) for 4 h. The mutagenized individuals were allowed to reproduce, and their offspring were then exposed to the same EMS mutagenesis protocol. This process was repeated an additional three times, ultimately producing the genetically variable CF3 population. The CF3 population was then exposed to heat-killed *S. marcescens* (Sm2170) for 30 passages and maintained at a population size of ~750 individuals, serving as a control in the Slowinski et al. (2016) study. Therefore, the CF3 population is a genetically variable obligately outcrossing *C. elegans* population that has been passaged in the lab for 30 generations after mutagenesis. The CF3 population was used as the obligate outcrossing ancestral host in this study.

#### Mixed-mating C. elegans populations

We backcrossed the wild-type mixed-mating allele back into the CF3 population to establish mixed-mating hermaphrodites to invade the obligately outcrossing CF3 population. To generate a population of mixed-mating *C. elegans* (WT CF3) that represented a genetic background within the CF3 population, CF3 females were mated with wild-type CB4856 males. Offspring capable of self-fertilization were backcrossed with obligately outcrossing nematodes for five generations. The resulting strain (WT CF3) was used as the ancestral mixed-mating population in this study.

### Bacterial strains

We chose the parasite *S. marcescens* because it is naturally occurring and capable of killing *C. elegans* after consumption and establishment of infection in the digestive tract (Kurz & Ewbank, 2000; Kurz et al., 2003; Pradel et al., 2007). The following *S. marcescens* strains were selected because they vary in virulence on *C. elegans*: Db11 (low virulence), Sm933 (mid virulence), SmD1 (high virulence), and Sm2170 (high virulence). The *S. marcescens* strain Db11 was obtained from the Caenorhabditis Genetics Center at the University of Minnesota (Minneapolis, MN), while both Sm933 and SmD1 were obtained from Carolina Biological Supply Company (Burlington, NC). The strain Sm2170 was obtained from S. Katz at Rogers State University (Claremore, OK).

The avirulent *Escherichia coli* strain OP50 was used as a food source for the nematode hosts in all experiments.

### Measuring virulence of pathogen strains

The initial (ancestral) virulence of each *S. marcescens* strain was determined separately for the CF3 (obligately outcrossing) and the WT CF3 (mixed-mating) ancestral host populations. The virulence of each coevolved pathogen strain was measured again on their respective coevolved host populations at generations 12 and 22 to monitor virulence evolution over the course of the experiment. Bacterial virulence was measured using survival assays on *Serratia* selection plates (SSPs), as described in Morran et al. (2011). Assays were conducted by liquid transferring L4 *C. elegans* to the *S. marcescens* side of SSPs seeded with *E. coli* and *S. marcescens*, in a process that mirrored the experimental evolution transfer protocol. After 48 h, the live worms remaining on the plates were counted, and survival and mortality were estimated as follows:


$$\begin{array}{l} \begin{array}{lll} & Survival\;=\;\frac{\;\#\;\;\;alive\;\;\;(counted)}{\;\#\;\;\;plated\;\;\;(estimated)}\; & Mortality=1-Survival\; \end{array} \end{array}$$

**Figure 1.**
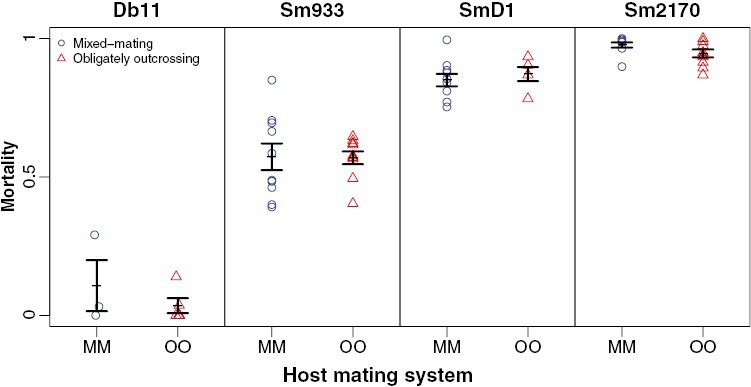
Mortality of the ancestral mixed-mating (blue circles) and obligately outcrossing (red triangles) host populations following 48-hr assays on each of the ancestral pathogen strains. Each point represents the mortality rate on a replicate assay plate (each plated with an estimated 165–189 worms). Error bars represent ±1 SEM. Mortality rates were unaffected by host mating system and were significantly different for all possible pairwise comparisons of pathogen strains.

**Figure 2.**
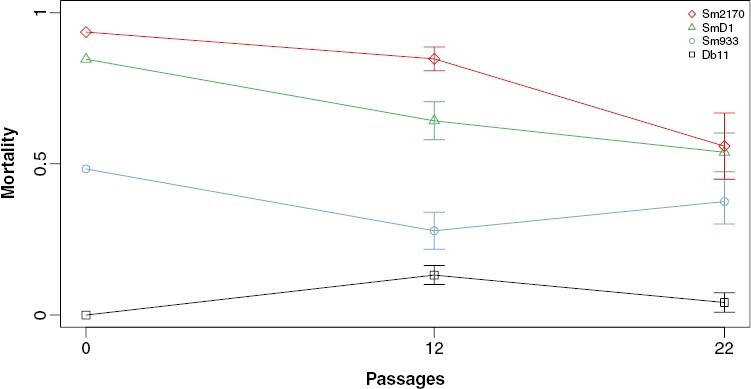
Average mortality (±1 SEM) of host populations assayed on the ancestral pathogens (passage 0) or on their contemporary coevolved pathogens (passages 12 and 22).

While the true population mortality must be greater than or equal to zero, our estimates of mortality are subject to sampling error (i.e., we plated worms by liquid transferring, and we estimated the number of worms plated based on the concentration of worms in solution and the volume of solution, but the exact number of worms plated may have differed slightly from our estimates). Consequently, some of our mortality estimates were slightly negative. For the purposes of our data presentation only, we constrained the plotted mortality scores to be greater than or equal to zero (i.e., we set mortality estimates that were less than zero equal to zero, and calculated mean and error bars using the bounded mortality rates). However, for the purposes of our hypothesis-testing statistical analyses, we used our raw estimates of mortality. Note that if we run our statistics on mortality scores bounded by zero we get qualitatively similar results (not shown). The treatment in which we observed some negative mortality estimates in the raw data (and in which we set negative mortality estimates to zero for the purposes of our data presentation) was the DB11 strain at generations 0, 12, and 22 (Figures 1 and 2).

### Virulence assay sample sizes

In the ancestral virulence assays, mortality was calculated on three to ten replicate assay plates for each host mating type on each parasite strain. In the coevolved virulence assays at generations 12 and 22, mortality was measured on three replicate assay plates for each host population at each time, and the average mortality rate across replicate plates was used as our estimate of population-level mortality rates. The estimated number of worms per liquid transfer varied by assay plate: 165 to 189 in the ancestral virulence mortality assays and 97 to 257 in the coevolved virulence mortality assays.

### *Serratia* selection plates

We conducted experimental evolution on SSPs. The protocol for preparing SSPs is described in Morran et al. (2011) and Slowinski et al. (2016) and was modified slightly for this study. SSPs were prepared by pouring 30 ml of autoclaved NGM Lite (US Biological, Swampscott, MA) into 10-cm Petri dishes. Petri dishes were then marked and divided laterally into three sections. The middle section of the dish remained unseeded, separating a section of the plate containing 60 μl of the *E. coli* strain OP50 from the remaining section, containing 35 μl of a *S. marcescens* parasite strain or population. The parasite side of each plate was seeded with one of the experimental *S. marcescens* strains (i.e., Db11, Sm933, SmD1, or Sm2170). Inocula were spread using sterile spreaders after bacterial solutions were first incubated overnight at 28 °C in test tubes of 5-ml lysogeny broth (LB). The plate was then incubated overnight at 28 °C prior to nematode exposure. After incubation, 40 μl of ampicillin (100 mg/ml) was streaked down the middle section of the plate to prevent worms from spreading *S. marcescens* into the *E. coli* lawns.

### Treatment groups

#### Fixed-parasite and heat-killed (control) parasite treatments

Inocula for the fixed-parasite treatment plates were produced using bacterial colonies taken from ancestral stock populations of each *S. marcescens* strain during each passage. The control parasite treatment plates were also generated using these ancestral stock populations, but, following their incubation in LB, bacteria were killed by exposing them to heat (80 °C) for 6 hr prior to administration to the plate.

#### Copassaged parasite treatment

The generation 0 SSPs for the copassaged parasite treatments were inoculated with the same ancestral stock populations of the *S. marcescens* strains that were used to inoculate fixed-treatment SSPs. Thereafter, parasites were passaged by first harvesting the bodies of 20 dead or morbid nematodes in the current SSPs. Once harvested and picked into 1 ml of M9 buffer, nematodes were centrifuged in solution at 3,000 RPM for one minute. After the supernatant was discarded, nematodes were rinsed five times with 1 ml of M9 buffer and then crushed. Bacterial cells were then streaked on NGM lite agar and incubated for 24 hr at 28 °C. A sample of 20 *Serratia* colonies was transferred to 5 ml of LB and grown overnight and subsequently used to seed the corresponding SSPs for the next passage. Thus, the copassaged treatment facilitated opportunities for reciprocal evolutionary change by imposing selection for both increased parasite virulence and for host defense.

### Passage of host populations for experimental evolution

Approximately 950 obligately outcrossing nematodes (strain CF3) and 50 mixed-mating nematodes (strain WT CF3) were transferred in M9 buffer onto the parasite treatment side of all plates at generation 0. After 4 days (approximately one host generation), all nematodes on the *E. coli* side of the SSPs plates were transferred off the plates. They were then washed three times with 1 mL M9 buffer and approximately 1,000 worms were then transferred to the next generation of SSPs. This protocol was repeated for 24 total host passages.

### Calculating selfing rates

Because the sex ratio of *C. elegans* offspring is directly determined by reproductive mode, with outcrossing producing a 1:1 ratio of males to hermaphrodites, while almost 100% of selfed offspring are hermaphrodites (Brenner, 1974) (or females if they express the obligate-outcrossing allele) the outcrossing rate and selfing rate in *C. elegans* populations can be accurately estimated based on male frequencies. The protocol for determining male frequencies, outcrossing rates, and selfing rates is described in Stewart & Phillips (2002) and Slowinski et al. (2016). Briefly, the sex of approximately 200 adult nematodes from a cross-section of the *E. coli* side of the SSPs was scored at passages 8, 16, and 24 to determine male frequency. Male frequency, outcrossing rates, and selfing rates were calculated as follows (Stewart & Phillips, 2002), assuming a nondisjunction rate of 0.0015 (Hodgkin & Doniach, 1997; Ward & Carrel, 1979):


$$\begin{array}{l} Male\;\;\;frequency=\frac{Males\;\;\;counted}{Total\;\;\;worms\;\;\;counted} \end{array}$$

$$\begin{array}{l} Outcrossing\;\;\;rate=\left(Male\;\;\;frequency\text{-}nondisjunction\;\;\;rate\right)*2 \end{array}$$

$$\begin{array}{l} Self\;ing\;\;\;rate=1-Outcrossing\;\;\;rate\; \end{array}$$

The generation 0 selfing rate was assumed to be 0.05 for all populations, based on the starting frequencies of the transferred mixed-mating (starting frequency = 0.05) and obligately outcrossing worms (starting frequency = 0.95), and based on the assumption that outcrossing rates in the ancestral mixed-mating populations were negligible.

### Statistical methods

#### Statistical software

All statistical analyses were run in R version 4.1.1, using the statistical package “lmerTest” version 3.1.3 (Kuznetsova et al., 2017). Plots for data visualization were also generated in R.

#### Measuring ancestral S. marcescens virulence

We used a two-way ANOVA to test the effect of host breeding system (mixed-mating or obligately outcrossing) and *S. marcescens* strain on host mortality following 48 hr on SSP assay plates. To determine which ancestral *S. marcescens* strains differed significantly from each other in their virulence, we used Tukey’s honestly significant difference tests to compare mortality rates across all possible pairwise comparisons of *S. marcescens* strains.

#### Coevolved virulence over time

To assess how the virulence of each *S. marcescens* population changed over the course of the experiment, at passage numbers 12 and 22 each coevolving host population was assayed on its contemporary coevolving *S. marcescens* population, and host mortality was measured following 48 hr on SSPs. We used a linear mixed-effects model (LMM) to test whether *S. marcescens* strain, passage number, and strain by passage interaction affected host mortality rates (Each estimate of a host population mortality rate was the mean of the mortality rates on three replicate assay plates.). Because host populations were sampled repeatedly across time points, replicate host population was treated as a random effect.

Because our LMM found a significant main effect of *Serratia* strain on mortality, we ran follow-up ANOVAs on mortalities at passage numbers 12 and 22. We used Tukey’s post hoc tests across all pairwise comparisons to determine which *Serratia* strains differed significantly from each other at each time point.

#### Selfing rates

We analyzed the data separately from each of the experimental evolution treatments (i.e., separate analyses for heat-killed, fixed, and coevolved data sets). Within each experimental evolution treatment, we ran an ANOVA at passage 8 to determine whether parasite strain affected the evolution of selfing rates during the first eight passages of experimental evolution. Then we ran a second ANOVA to determine whether differences in selfing rates across parasite strains were maintained to passage 24. In each case, if we found a significant effect of parasite strain on selfing rates, then we used post hoc Tukey’s HSD tests across all possible pairwise comparisons of parasite strains to determine which parasite strain pairs differed significantly in their impact on hosts’ selfing rates.

## Results

### Virulence of ancestral *S. marcescens* strains

Ancestral *S. marcescens* strains varied significantly in their virulence when assayed on ancestral hosts (*F*_3_ = 180, *P* < 0.001; Figure 1). Mortality rates were not different between obligately outcrossing and mixed-mating ancestral hosts on the ancestral parasite (*F*_1_ = 0.868, *P* = 0.355). Tukey’s post hoc comparisons revealed that mortality was significantly different in all possible pairwise comparisons between *S. marcescens* strains. *P*-values for all Tukey’s post hoc tests are found in Table 1.

**Table 1.** *p-*Values for pairwise comparisons between *S. marcescens* parasite strains, as determined by Tukey’s HSD tests (described in Methods). Each row represents pairwise comparisons between the strains indicated in the first two columns. Virulence of each strain is indicated in parentheses. Experimental timepoint and trait being compared are indicated in column headings. Significant pairwise differences (*p* < .05) indicated in bold. Note that main effects of parasite strains are reported in the body of the results section and are not included in the table. Post hoc pairwise comparisons were only run for traits and time points for which there was a significant effect of parasite strain. Traits that were not significantly affected by parasite strain (e.g., selfing rates at all time points in the heat-killed treatment) are not included in the table.

Strain 1	Strain 2	Virulence of ancestral strains: generation 0	Coevolved virulence: passage 12	Coevolved virulence: passage 22	Selfing rates: passage 8 (fixed treatment)	Selfing rates: passage 8 (copassaged treatment)	Selfing rates: passage 24 (copassaged treatment)
Db11 (low)	Sm933 (mid)	***p* < .001**	*p* = .212	*p* **= .010**	*p* = .906	*p* = .937	*p* = .787
Db11 (low)	SmD1 (high)	***p* < .001**	***p* < .001**	***p* < .001**	***p* < .001**	***p* = .015**	***p* < .001**
Db11 (low)	Sm2170 (high)	***p* < .001**	***p* < .001**	***p* < .001**	***p* = .006**	***p* = .001**	***p* < .001**
Sm933 (mid)	SmD1 (high)	***p* < .001**	***p* < .001**	*p* = .322	***p* = .002**	*p* = .051	***p* = .002**
Sm933 (mid)	Sm2170 (high)	***p* < .001**	***p* < .001**	*p* = .319	***p* = .018**	***p* = .004**	***p* < .001**
SmD1 (high)	Sm2170 (high)	***p* = .006**	*p* = .086	*p* = .997	*p* = .560	*p* = .423	*p* = .485

### Coevolved virulence over time

We found a significant main effect of *Serratia* strain (*F*_3_ = 15.9, *P* < 0.001) on host mortality, indicating that virulence varied significantly across parasite strains. We also found a significant main effect of passage number (*F*_1_ = 6.76, *P* = 0.013) on host mortality, indicating that parasite virulence declined over the course of the experiment on coevolved populations (Figure 2). This pattern seemed to be primarily driven by declining virulence of the high virulent parasite strains; however, there was no significant *Serratia* strain by passage number interaction on host mortality (*F*_3_ = 2.378, *P* = 0.084).

We found a significant effect of *Serratia* strain on mortality at both passage 12 (*F*_3_ = 35, *P* < 0.001) and passage 22 (*F*_3_ = 12.46, *P* < 0.001) using ANOVAs. Post hoc tests (*P*-values in Table 1) at passage 12 revealed that mortality on Db11 was still significantly lower than mortality on SmD1 and Sm2170. Mortality on Sm933 was also still significantly lower than on SmD1 and Sm2170. However, there was no longer a difference in mortality between Db11 and Sm933 or between SmD1 and Sm2170. Post hoc tests also revealed that the Db11 strain was still significantly less virulent than all other strains at passage 22. However, no pairwise comparisons between the parasite strains Sm933, SmD1, and Sm2170 were significant at passage 22.

Importantly, none of the lines designating the virulence of *S. marcescens* strains over time crossed, indicating that the rank order of strain virulence was relatively stable over the course of experimental coevolution (Figure 2).

### Selfing rates

**Figure 3.**
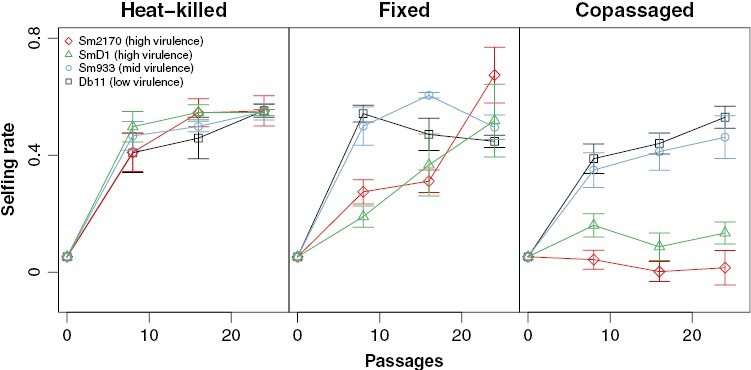
Selfing rates over time measured in *C. elegans* host populations evolving on heat-killed (control; left), fixed (nonevolving; middle), or copassaged (right) *S. marcescens* strains. Within each passaging treatment, replicate host populations evolved on the Db11 (low virulence, black square), Sm933 (mid virulence, blue circle), SmD1 (high virulence, green triangle), or Sm2170 (high virulence, red diamond) strain of *S. marcescens*. Error bars represent ± 1 SEM.

In the heat-killed (control) treatment, host selfing spread at a similar rate on all the parasite strains (Figure 3). There was no significant effect of parasite strain on passage 8 selfing rates (*F*_3_ = 0.552, *P* = 0.656) or on passage 24 selfing rates (*F*_3_ = 0.009, *P* = 0.999).

In the fixed treatment, we found a significant effect of parasite strain on passage 8 selfing rates (*F*_3_ = 14.5, *P* < 0.001, Figure 3). Pairwise comparisons (*P*-values in Table 1) revealed that selfing spread faster during the first eight passages of selection in the low- (Db11) and mid-virulence (Sm933) strains than in the high-virulence (SmD1 and Sm2170) strains. There was no difference in passage 8 selfing rates between the low- and mid-virulence strains (Db11 vs. Sm933) or between the two high-virulence strains (SmD1 vs. Sm2170). In the fixed treatment, there was no significant effect of parasite strain on passage 24 selfing rates (*F*_3_ = 1.44, *P* = 0.28), indicating that differences in selfing rates across parasite strains observed early in the experiment were lost by the end of the experiment.

In the copassaged treatment, there was a significant effect of parasite strain on passage 8 selfing rates (*F*_3_ = 9.7, *P* < 0.001, Figure 3). Pairwise comparisons (*P*-values in Table 1) revealed that selfing rates at passage 8 were higher on the low- (Db11) and mid-virulence (Sm933) parasite strains than on the high-virulence parasite strains SmD1 and Sm2170. However, selfing rates at passage 8 did not differ between the low- (Db11) and mid-virulence (Sm933) strains or between the high-virulence strains (SmD1 vs. Sm2170). There were still differences in selfing rates across parasite strains at passage 24 (*F*_3_ = 19.96, *P* < 0.001). At passage 24 the low- (Db11) and mid-virulence (Sm933) parasite strains still maintained significantly higher selfing rates than the high-virulence strains (SmD1 and Sm2170). Selfing rates between the low- (Db11) and mid-virulence (Sm933) strains still did not differ significantly at passage 24 nor did they differ significantly at passage 24 between the two high-virulence strains (SmD1 and Sm2170).

## Discussion

Theory predicts that for a parasite to maintain high host outcrossing, it must be coevolving with the host and highly virulent. We tested the prediction that only highly virulent coevolving parasites could constrain the spread of self-fertilization within outcrossing host populations. As predicted, self-fertilization was able to invade *C. elegans* host populations in all heat-killed (control) and fixed (nonevolving) parasite treatments, regardless of parasite virulence. In the copassaged parasite treatments, however, self-fertilization was able to invade when hosts were copassaged with low- (Db11) or mid-virulence (Sm933) parasite strains but was unable to invade host populations that were copassaged with the highly virulent pathogen strains SmD1 and Sm2170 (Figure 3). Taken together, our results suggest that parasites can maintain elevated levels of biparental sex in host populations, but only when parasites are both highly virulent and coevolving.

The rapid invasion and spread of self-fertilization in the heat-killed (control) populations occurred as predicted by theoretical models of the costs of biparental sex (Agrawal & Lively, 2001; Charlesworth, 1980; Fisher, 1941; Maynard Smith, 1978). Additionally, the delay in the invasion of self-fertilization within obligately outcrossing host populations exposed to highly virulent parasites (SmD1 and Sm2170) in the fixed-parasite treatment was consistent with previous work (Morran et al., 2011; Slowinski et al., 2016). This delay in the spread of selfing into host populations exposed to nonevolving virulent parasites suggests that a nonevolving parasite can produce a novel selective environment that can temporarily favor host outcrossing rates above control levels. However, nonevolving parasites did not maintain elevated host outcrossing rates long term relative to the control, suggesting that outcrossing is no longer selected for after the host adapts to its nonchanging parasite environment.

Our results provide strong evidence that virulent coevolving parasites can maintain host outcrossing. However, the mode of selection imposed by parasites within our experiments is still unclear. Thus, our results support the RQH, but are not a comprehensive test. While our results generally support the RQH, they also demonstrate potential limitations in the ability of parasites to select for biparental sex. Given that the spread of self-fertilization occurred in all host populations except for those that were coevolving with highly virulent parasite strains, both coevolution and high virulence may be necessary for a parasite to maintain host outcrossing.

While the RQH on its own may only be able to explain the maintenance of outcrossing in cases of high parasite virulence, that does not preclude parasite-mediated selection from being a critical part of a general explanation for the maintenance of sex and outcrossing in nature. Indeed, other factors like inbreeding depression or Hill–Robertson interference (Hill & Robertson, 1966) may impose additional selection favoring the maintenance of outcrossing (Hartfield & Keightley, 2012) and could act in synergy with parasite-mediated selection (da Silva & Galbraith, 2017; Hodgson & Otto, 2012) to maintain outcrossing. As predicted by the pluralist approach to explain the maintenance of sex, selection imposed by deleterious mutations acting synergistically with parasite-mediated frequency-dependent selection may serve as a broad explanation for outcrossing by lowering the threshold level of parasite virulence necessary to maintain host outcrossing (Howard & Lively, 1994, 1998; Neiman et al., 2017; West et al., 1999).

Additionally, while one coevolving parasite population of low- to mid-virulence was not sufficient to maintain host outcrossing, perhaps the combined effects of multiple low- to mid-virulence parasites simultaneously coevolving with a host population could maintain outcrossing. Coinfection with multiple parasites is common in plants and animals (Brogden, 2002; Hood, 2003; Petney & Andrews, 1998; Stirnadel & Ebert, 1997), as reviewed in Mostowy et al. (2010). Under some circumstances, the addition of a second parasite to a host–parasite model can lead to significantly more sex than predicted based on a single parasite model (Mostowy et al., 2010). Finally, additional other selective pressures may act synergistically with parasite-mediated selection to expand the conditions under which outcrossing is favored by selection. Overall, while coevolving parasites alone may not always be sufficient to explain the maintenance of outcrossing, they may be an important factor contributing to mating system evolution in many systems.

## Data Availability

Data and code for our statistical analyses are available on Dryad at https://datadryad.org/stash/dataset/doi:10.5061/dryad.jm63xsjh9.
